# Nine years experience in surgical approach of leiomyomatosis of esophagus

**DOI:** 10.1186/1477-7819-7-102

**Published:** 2009-12-23

**Authors:** Christos Asteriou, Dimitrios Konstantinou, Miltiadis Lalountas, Athanassios Kleontas, Konstantinos Setzis, Georgios Zafiriou, Nikolaos Barbetakis

**Affiliations:** 1 CardioThoracic Surgery Department, Theagenio Cancer Hospital, Al. Symeonidi 2, Thessaloniki, 54007, Greece; 2 2nd Department of Chemotherapy, Theagenio Cancer Hospital, Al. Symeonidi 2, Thessaloniki, 54007, Greece; 3 2nd Propedeutical Department of Surgery, Aristotle University of Thessaloniki, Hippokratio General Hospital, Konstantinoupoleos 49, Thessaloniki, Greece; 4 1st Department of Surgery, Theagenio Cancer Hospital, Al. Symeonidi 2, Thessaloniki, 54007, Greece

## Abstract

**Background:**

Leiomyomas of esophagus, although rare, are the most frequent benign tumors of esophagus. Aim of this study is the presentation of 7 patients with esophageal leiomyomas who underwent surgical treatment during a 9-year period.

**Methods:**

Epidemiological data (sex, age), the presenting symptoms, diagnostic examinations, tumor location, histopathological findings and the safety and efficacy of surgical resection are analyzed and assessed.

**Results:**

5 men and 2 women with mean age of 56.9 years were operated. In 3 cases the tumor was located at the lower esophagus, while in the other 4 cases, the leiomyoma was found at the median third of esophagus. 4 patients had severe symptoms related to the leiomyoma, such as dysphagia and epigastric pain. All patients underwent a right postolateral thoracotomy with enucleation of the lesion. None of them received resection of part of the esophagus. The mean diameter of the resected tumors was 4.3 cm. The dimensions of leiomyomas were immediately associated with the symptoms. In no case was detected malignancy or recurrence. All patients were relieved from their symptoms, while postoperative morbidity and mortality did not occur.

**Conclusions:**

Esophageal leiomyoma is a benign tumor, which causes symptoms only if its size becomes large. Surgical enucleation is considered to be safe and effective, without complications.

## Background

The esophageal leiomyoma is a benign tumor of the esophagus. Other non-malignant lesions of esophagus are hemangioma, lymphangioma, squamous papilloma, fibrovascular polyp and granular cell myoblastoma. The incidence of this kind of lesions is referred to be almost 1% of the esophageal neoplasms in the international literature [1]. Leiomyomas are the most common benign tumors of esophagus. The main symptoms usually are dysphagia and epigastric pain, but they are not specific for the disease. Differential diagnosis should always include esophageal cancer [2]. It is important for the modern cardiothoracic surgeon to be aware of this entity. Here, a small case series of 7 patients who were treated in our Institute during the last 9 years is presented, with emphasis in diagnosis and management.

## Patients and Methods

This study is a retrospective analysis of the medical records of patients, whose diagnosis was a possible esophageal leiomyoma. The study took place at the Theagenio Cancer Hospital of Thessaloniki from September 2000 to September 2009. Seven patients were detected. The epidemiological data (sex, age), presenting symptoms, diagnostic examinations, tumor location, histopathological findings and the safety and efficacy of the surgical resection were analyzed. The standard examinations included preoperative esophagogastroscopy, endoscopic ultrasonography and computed tomography of the chest. Fine needle aspiration was not performed in any case.

## Results

Patients' group was consisted of five males and two females. Their age ranged from 48 to 67 years (mean age: 56.9 years). The most common symptoms were dysphagia and epigastric pain, which were present in four cases. In addition, one patient was complaining for retrosternal burnings. The rest of the cases had limited symptomatology, like unspecified discomfort located at the chest or the upper abdomen. All patients were subjected to the standard examinations, which demonstrated an esophageal tumor with features compatible with leiomyoma. In three cases the tumor was located at the lower third of the esophagus, while four lesions were detected at the median third. In all cases a right postolateral thoracotomy carried out and myotomy of esophagus with enucleation of the neoplasm took place. Frozen sections showed typical leiomyoma of esophagus. None of the patients underwent resection of part of the organ. The mean diameter of the resected tumors was 4.3 cm. Malignancy or recurrence was not detected. The mean in-hospital staying was 7 days. Complications did not occur. All patients were relieved from their symptoms, after surgical removal of the tumor. Postoperative follow-up did not reveal any morbidity or mortality related to the primary diagnosis. Clinical presentation, diagnostic findings and management of the patients are summarized in table 1.

**Table 1:** Clinical presentation, diagnostic examinations' results and surgical approach.

Sex	Age	Symptoms	EGS-EUS	Diameter	Treatment
♂	67	Dysphagia	Submucosal hypoechoic tumor at 22-28 cm	5,9 cm	Right Thoracotomy-Enucleation

♂	48	Epigastric discomfort	Submucosal hypoechoic nodule at 29-32 cm	2 cm	Right Thoracotomy-Enucleation

♂	51	Epigastric pain, Dysphagia	Submucosal hypoechoic tumor at 23-28 cm	4,8 cm	Right Thoracotomy-Enucleation

♂	59	Epigastric discomfort	Submucosal hypoechoic nodule at 22-26 cm	2,7 cm	Right Thoracotomy-Enucleation

♂	57	Dysphagia	Submucosal hypoechoic tumor at 27-33 cm	5,2 cm	Right Thoracotomy-Enucleation

♀	61	Retrosternal burning, Dysphagia	Submucosal hypoechoic tumor at 20-27 cm	6,5 cm	Right Thoracotomy-Enucleation

♀	55	Epigastric discomfort	Submucosal hypoechoic nodule at 29-34 cm	3,1 cm	Right Thoracotomy-Enucleation

EGS: Esophagogastroscopy, EUS: Endoscopic Ultrasonography

## Discussion

Leiomyomas belong to benign mesenchymal tumors of esophagus. They are the most common non-malignant lesions of esophagus, with an incidence approaching 60% of all benign tumors of the organ [1]. The symptoms accompanying esophageal leiomyomas are not specific. It seems that the size of tumor correlates with the severity of the symptoms. Dysphagia with concomitant epigastric pain or retrosternal burning usually appears when the tumor's diameter becomes larger than the critical point of 4.5-5 cm [3]. Smaller leiomyomas may cause mild symptomatology, like unspecified discomfort, or even may be asymptomatic at all. In the majority of cases the lesions are located at the distal two thirds of the esophagus. In our small series, the distribution was almost equal in the two aforementioned positions.

**Figure 1:**
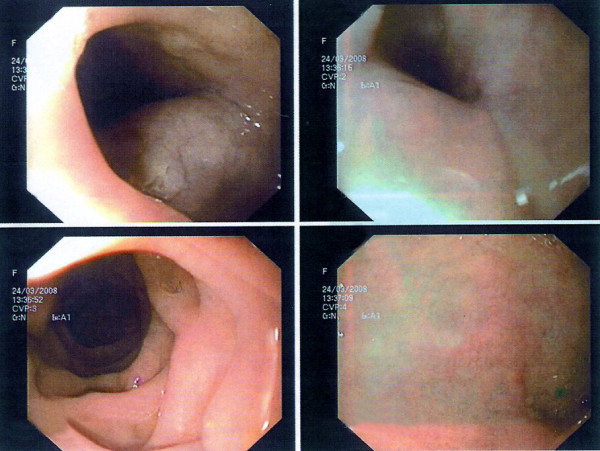
**Endoscopic view of an esophageal leiomyoma located at the median third**. A submucosal lesion compressing the lumen of esophagus, which however is leaving intact the overlying mucosa, is demonstrated.

**Figure 2:**
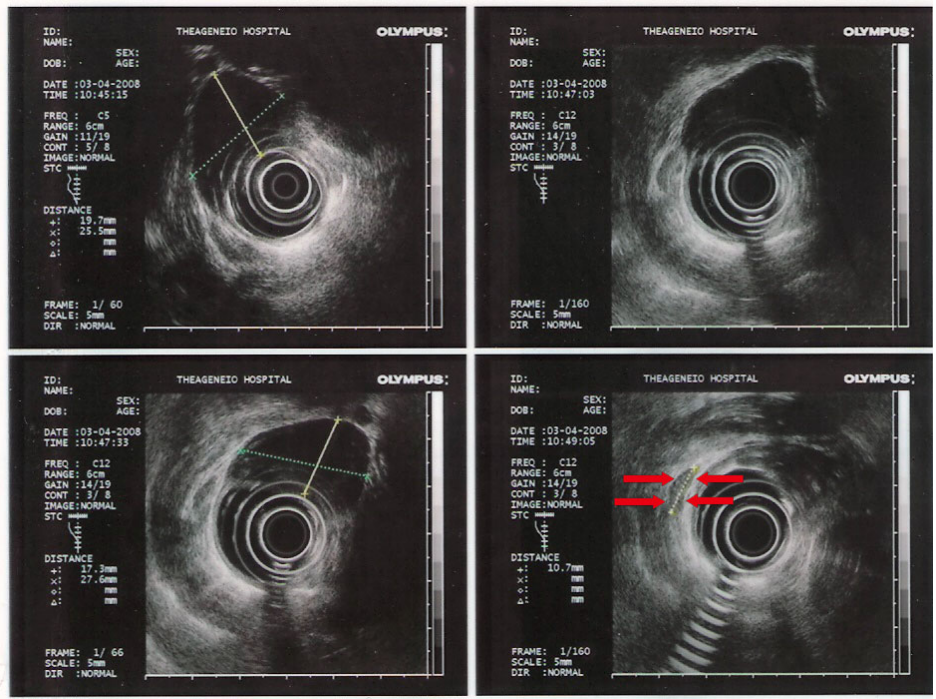
**Endoscopic ultrasonographic evaluation of a leiomyoma**. The tumor is presented as a well-demarkated, homogeneous and hypoechoic lesion with clear margin, originating from muscularis mucosa. In this case, its size is 2.7 × 1.7 cm. A small lymph node (1.07 cm) is also discovered (red arrows).

**Figure 3:**
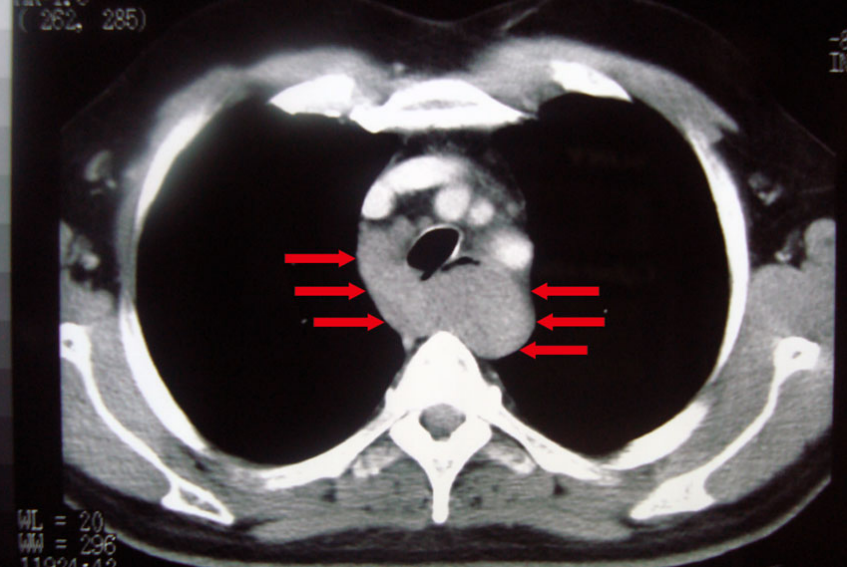
**Computed Tomography of the chest revealing a large mass originating from median esophagus (red arrows)**.

Leiomyomas can mimic cancer of esophagus. Lack of specific symptoms as well as the similarity in initial clinical expression may cause diagnostic confusion. It is, therefore, obligatory the full preoperative investigation of each patient complaining for symptoms possibly relating with an esophageal lesion. Esophagogastroscopy combined with endoscopic ultrasonographic evaluation of the tumor is mandatory in order to exclude cancer of esophagus from the differential diagnosis [4,5]. Leiomyoma's typical appearance is of homogeneous and hypoechoic lesion with clear margin (Fig 1, 2) [6-8]. Computed Tomography scans of the chest ideally complete the preoperative evaluation of the patients, revealing in most cases a mass originating from esophagus without mediastinal lymphadenopathy (Fig 3). Preoperative biopsy of the tumor is a debating issue [9]. Our policy is not to recommend it, because an esophageal leak or fistula can occur with a risk of potential mediastinitis. Moreover, in many cases fine needle aspiration could not provide enough material to establish an accurate histopathological diagnosis. The high risk of complications in combination with the small benefit for the patient suggests not to perform this diagnostic procedure, although other investigators recommend Fine Needle Biopsy via endoscopic ultrasonography.

Every symptomatic leiomyoma should be excised. In case the tumor is discovered accidentally, some authors recommend regular follow-up with barium swallow and endoscopy [9]. Our policy is that a surgical removal is recommended even in this situation, because there is always the possibility, rarely though, of malignant transformation.

Different kinds of approaches have been described depended from the location of the tumor. In the majority of the cases the tumor is discovered at the mean or the distal third of the esophagus. Right thoracotomy is suggested in first case; while a left thoracoabdominal approach fits better the second case [10]. Our experience shows that using a right postolateral thoracotomy, excision of the lesion is feasible in both locations. Myotomy of esophagus and extramucosal enucleation of the leiomyoma is the standard and established procedure. The external muscular layer of esophagus is incised longitudinally. Dissection and excision of the tumor with great care not to open the mucosa completes the surgical procedure. If mucosa is penetrated, careful reapproximation with absorbable sutures takes place, while closure of the muscle layers is obligatory, in order to prevent leak. A lung or pleural flap graft may be used in order to seal a potential leakage. In very few cases resection of part of esophagus is described for large tumors [2,10]. In our opinion, enucleation of the tumor is the only indicated surgical approach of the leiomyoma. Esophagogastrectomy remains the operation of choice exclusively when dealing with esophageal cancer. It is a major operation with concomitant morbidity and mortality due to its possible severe complications.

**Figure 4:**
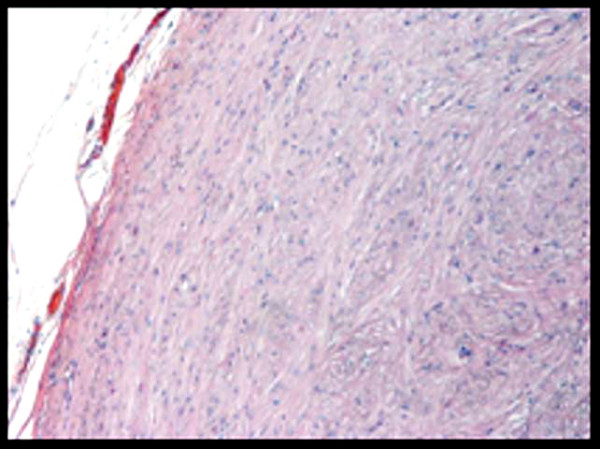
**Histopathological view of leiomyoma (H-EX100)**. The tumor is composed of clusters and bundles of elongated cells with ovoid nuclei and varying amounts of eosinophilic fibrillar cytoplasm.

Histopathologicaly, the tumor is composed of bland spindle cells and demonstrates low to moderate cellularity. The cells have eosinophilic and fibrillary cytoplasm (Fig 4). Mitotic figures are rare. Spherical calcifications are also focally present. Leiomyomas are presented typically globally positive for desmin and smooth muscle actin, while they are negative for CD34 and CD117 (c-kit) [11]. Differential diagnosis from esophageal cancer (squamous cell carcinoma of esophagus or adenocarcinoma of gastroesophageal junction) should not be a problem.

## Conclusions

In conclusion, leiomyoma is a rare benign tumor of esophagus. Correct preoperative evaluation is of great importance in planning of the surgical excision. Enucleation of the lesion using a right postolateral thoracotomy is the most common approach. Postoperative complications are rare, while morbidity and mortality rates tend to be zero universally. Patients' relief from the symptoms is the rule and the prognosis is expected great.

## Competing interests

The authors declare that they have no competing interests.

## Authors' contributions

CA, DK, ML, AK, KS, and GZ took part in the care of the patients and contributed equally in carrying out the medical literature search and preparation of the manuscript. NB participated in the care of the patients and had the supervision of this report. All authors approved the final manuscript.

## Consent

Written informed consent was obtained from all patients for publication of this article and accompanying images. Copies of the written consents are available for review by the Editor-in-Chief of this journal.
